# Census Bulletin No. 6

**Published:** 1890-11

**Authors:** 


					﻿Census Bulletin No. 6. Robert P. Porter, Superintendent
of the Census.
This bulletin deals with the financial condition of all the counties of every
State and Territory in the Union. It is a pamphlet of twenty-six pages, full
of formidable tables and interspersed with maps of the various sections of the
United States, showing at a glance just what the indebtedness and where
located.
These bulletins are certainly valuable to all who are interested in the gen-
eral prosperity of this nation.	W. M. J.
				

